# The next age of immunotherapy: optimisation, stratification and therapeutic synergies

**DOI:** 10.1038/s41416-018-0330-4

**Published:** 2018-11-09

**Authors:** Niels Halama

**Affiliations:** National Center for Tumor Diseases (NCT) and Internal Medicine VI and Institute for Immunology, University Hospital Heidelberg, Heidelberg, Germany

## Abstract

Cancer immunotherapy has entered a phase of broad application in the treatment of patients with haematologic and solid tumours. From first steps to standard of care, immunotherapy has established its utility and applicability across different cancer types. Now it must demonstrate its higher potential in more personalised and stratified approaches.

## Main

Ten to fifteen years ago, clinical tumour immunology was mainly regarded as an esoteric scientific playground for enthusiasts. Clinical developments in the ensuing years have progressed so dramatically that immunological therapies have become the standard of care for many advanced types of cancer. This change from an area driven by fundamental research into a translational arena with a plethora of new therapies, side effects, and needs for novel biomarkers is truly a transformative one for the field of oncology. Despite these developments, clinical translation is often limited by our understanding of the tight network and regulation of immune responses in all parts of the human body. Our knowledge of the key pathways and counter-mechanisms involved in immune responses needs to be improved to harness the full potential of immunotherapy. The question is no longer ‘Can the immune system be used as a potential tool in the fight against cancer?’ but, instead, ‘What are the key regulators required to achieve prolonged success with immunotherapies for specific cancers?’. This special issue on immunotherapy aims to address these fundamental questions, with articles providing valuable insight into immunotherapy design, stratification and toxicity management.

## The immune microenvironment and patient stratification

Fundamental studies on the immunological microenvironment of tumours have shed light on the relationship between the composition of immune cells and a patient’s clinical course. With this deeper knowledge of the immune contexture in different cancer entities, Sanchez-Salaz et al.^1^ discuss critical components of the tumour microenvironment, including the immune cell composition, and highlight the importance of assessing the tumour immune microenvironment as a whole to best predict patient prognosis. The adaptive arm of the immune system has classically received most attention in this regard, with its prominent potential for prognostication and therapeutic management. Here, Groth et al.^2^ take a look at the role of the innate arm of the immune system, with a specific focus on the myeloid-derived suppressor cells. Neither the inhibitory potential of these cells nor their therapeutic potential in the clinic is yet fully clear. Still focussing on a key component of innate immunity in cancer, the role of neutrophils in counteracting the effects of anti-vascular endothelial growth factor (VEGF) therapy in colorectal cancer is presented by Fritsch et al.^3^ The complexity in translating results from basic research and clinical trials into a framework of personalised medicine poses a challenge; progressing from making relevant findings in the microenvironment to treatment decisions is still immensely difficult, and there are ample areas for development.

## Cytokines, receptors, checkpoints… and a Nobel prize

Cytokines have long been used in multiple clinical settings, with varying degrees of success; Sanmamed et al.^4^ put together an elegant overview of the current status of cytokine-based immunotherapies, and key concepts that must be considered for their future development. Furthermore, in pioneering new research, an involvement of histamine receptor H4 in regulating anti-tumour immunity has been elucidated in the work by Sterle et al.^5^ The discovery of immune checkpoints, particularly those mediated by programmed cell death 1 (PD-1) and cytotoxic T-lymphocyte–associated antigen 4 (CTLA-4), which recently earned James P. Allison and Tasuku Honjo the Nobel Prize in Physiology or Medicine, heralded a new era in cancer immunotherapy. While the expression of PD-1 and PD-L1 in tissues is being used as a parameter to stratify patients (the latter is currently already used in lung carcinoma), Hirsch et al. ^6^ explore the overall landscape of histological cancer types that are receptive to inhibition by the PD-1–PD-L1 axis in the next generation of combination immunotherapies. The combined use of immune checkpoint inhibitor therapies results in higher toxicity. The management of patients with (autoimmune) side effects from immunotherapies is challenging and leads to clinically difficult situations (including cytokine storm, pseudo-progression, severe autoimmune phenomena, etc.). Focussing on the normal immune system rather than immune dysregulation within the tumour, Khan et al.^7^ report on a new approach to identify patients who are at risk of developing immune-related side effects before undergoing immunotherapy. The authors analysed pre-existing/underlying systemic  immune dysregulation, which, similar to pre-therapeutic stratification, could lead to improved patient care and enhance the safety profile of immunotherapies.

## Emerging therapeutic opportunities

The next generation of immunotherapies involves the use of genetically modified T cells—chimeric antigen receptor T cells (CAR T cells)—as therapeutic agents. This technology has shown dramatic clinical benefit in the treatment of haematologic malignancies and promises a situation for multiple myeloma in which CAR T cell therapy might evolve into a standard treatment in the next years, as Kriegsmann et al.^8^ report. CAR T cell therapy for solid tumours has not so far shown promising results, but efforts to engineer CAR T cells with increased potency are ongoing. Technical aspects of the design of genetically engineered T cells as therapeutics are discussed by Tokarew et al.,^9^ who outline a roadmap of hurdles and possible developments for the successful use of these CAR T cells to treat many more cancer entities, focussing on tumour recognition, infiltration, persistence and resistance to suppressive signals in the microenvironment.

Another therapeutic approach involves targeting ‘private’ neoantigens—antigens found only on the surface of cancer cells—in a given patient. Meng et al.^10^ screened tumour-infiltrating lymphocytes from pancreatic cancer to identify T cell targets; the authors show a way to identify robust T cell targets and facilitate rapid translation into improved individualised immunotherapies, even in an ‘immunologically difficult’ entity like pancreatic cancer.

## Outlook

The next age of immunotherapy will be heralded by a deeper understanding of the effects of therapies and of the immunological microenvironment. The translation of personalised approaches into clinical therapies is rapidly becoming a reality, in line with the identification of common (genetic, microbiomic and proteomic) immunological signatures as a means of stratification for specific immunotherapies. These approaches are complemented by the recognition that the innate arm of the immune system is a pivotal part of an effective immune response, or the key resistance player against successful immunotherapy. An increased understanding of the possible indicators of side effects of immunotherapies will lead to improved clinical care and make the application of immunotherapies far safer for patients. The manuscripts presented in this special issue are a clear indicator of the next age of immunotherapy—an age of personalised immunotherapy in which more and more ‘uncharted lands’ are disappearing from the immunological treatment map.
